# Biomass-Based Polymer Nanoparticles With Aggregation-Induced Fluorescence Emission for Cell Imaging and Detection of Fe^3+^ Ions

**DOI:** 10.3389/fchem.2020.00563

**Published:** 2020-07-03

**Authors:** Shiyan Han, Jiaxin Ni, Youqi Han, Min Ge, Chunlei Zhang, Guiquan Jiang, Zhibin Peng, Jun Cao, Shujun Li

**Affiliations:** ^1^Postdoctoral Station of Mechanical Engineering, Northeast Forestry University, Harbin, China; ^2^Key Laboratory of Bio-Based Material Science and Technology (Northeast Forestry University), Ministry of Education, Harbin, China; ^3^Key Laboratory of Wooden Materials Science and Engineering of Jilin Province, Beihua University, Jilin, China; ^4^Department of Orthopedic Surgery, The First Affiliated Hospital of Harbin Medical University, Harbin, China

**Keywords:** larch bark, polymeric nanoparticles, aggregation-induced emission, cellular imaging, fluorescent probe for Fe^3+^

## Abstract

Polymeric nanoparticles, which show aggregation-induced luminescence emission, have been successfully prepared from larch bark, a natural renewable biomass resource, in a simple, rapid ultrasonic fragmentation method. The structure, element, particle size and molecular weight distribution of larch bark extracts (LBE) were studied by FTIR, XPS, TEM, XRD and linear mode mass spectrometry, respectively. LBE was found containing large numbers of aromatic rings, displaying an average particle size of about 4.5 nm and mainly presenting tetramers proanthocyanidins. High concentration, poor solvent, low temperature and high viscosity restricted the rotation and vibration of the aromatic rings in LBE, leading to the formation of J-aggregates and enhancing the aggregation-induced fluorescence emission. LBE possessed good resistance to photobleaching under ultraviolet light (200 mW/m^2^). Cytotoxicity experiments for 24 h and flow cytometry experiments for 3 days proved that even the concentrations of LBE as high as 1 mg/mL displayed non-toxic to MG-63 cells. Therefore, LBE could be employed for MG-63 cell imaging, with similar nuclear staining to the DAPI. The effects of different metal ions on the fluorescence emission intensity of LBE were analyzed and exhibited that Fe^3+^ owned obvious fluorescence quenching effect on LBE, while other metal ions possessed little or weak effect. Furthermore, the limit of detection (LOD) of Fe^3+^ was evaluated as 0.17 μM.

## Introduction

**Scheme 1:**
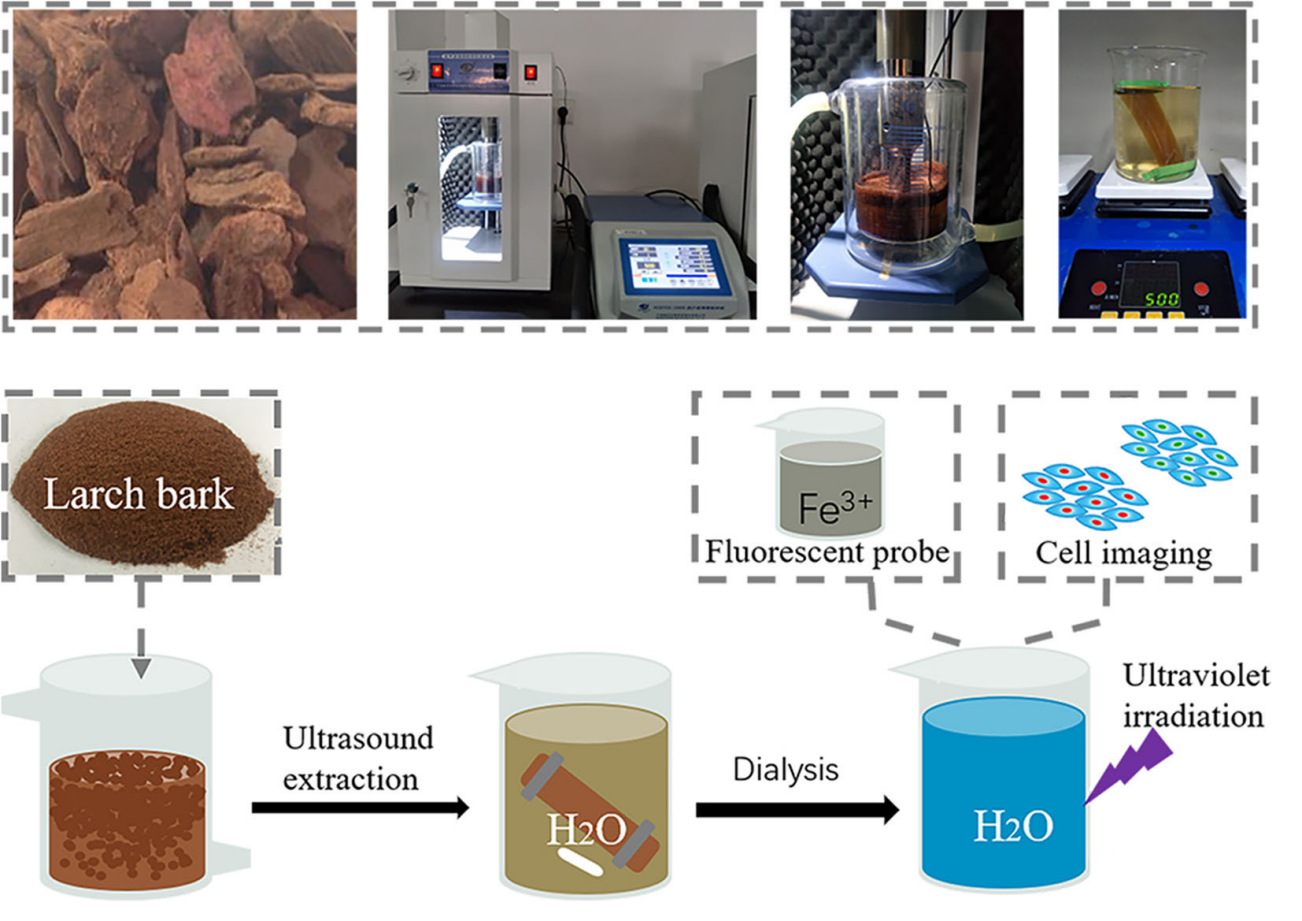
Preparation of fluorescent polymeric nanoparticles.

Fluorescent nanoparticles, including organic molecules (Lou et al., 2016; Zhang et al., 2017) graphene quantum dots (Zhu et al., 2016) and carbon dots (Wang et al., 2014; Chang et al., 2019), possess low toxicity and good biocompatibility, and are resistant to photobleaching, which exhibit enormous potential in cellular and *in vivo* imaging (Wang et al., 2013; Ding et al., 2016, 2018; He et al., 2018), as fluorescent probes (Qu et al., 2013; Zhang et al., 2014; Li et al., 2019a; Qi et al., 2019), in light-emitting diodes (Chiang et al., 2005; Lee et al., 2016; Tachibana et al., 2017; Liu et al., 2018; Wu et al., 2019), as ultraviolet and blue light blockers (Park et al., 2019) and for the detection of counterfeit materials (Chen et al., 2017). At present, organic molecules fluorescent nanoparticles are most commonly used in the field of cellular imaging and fluorescent probes. Especially researches into organic molecule-based nanoparticles with aggregation-induced emission (AIE) in cellular imaging have made especially good progress, duo to high selectivity, high sensitivity and good biocompatibility (Hu et al., 2014; He et al., 2018). Better AIE performance can be achieved with polymeric molecules than that with small organic molecules because the structure, morphology and function of the polymers can be fine-tuned, thus can be employed in cellular imaging and as fluorescent probes (Wang et al., 2013; Gu et al., 2016). Although much progress have been made in the study of fluorescent nanoparticles based on organic molecules, many challenges remain still to be accomplished. Most small molecule-based and polymeric fluorescent nanoparticles require expensive and time-consuming synthesis and toxic reagents, which limit their wide applications. The preparation of polymeric nanoparticles with AIE property by simple extraction of renewable natural raw material would be very attractive, which was paid little concern. Biomass resources have been extensively studied because of their excellent properties such as wide sources, non-toxic, renewable and so on (An et al., 2019; Du et al., 2019; Li et al., 2019c), in addition, they are also used as raw materials for the preparation of fluorescent materials due to their own sustainability (He et al., 2018; Li et al., 2019b). Larch bark is typically regarded as waste, but larch bark extracts (LBE) contain large amounts of catechol-containing compounds (Jiang et al., 2014, 2016; Luo et al., 2019). In previous reports, LBE have many medicinal values and are used as a free radical scavengers, antioxidants and anti-tumor drugs (Sharma et al., 2010; Ouchemoukh et al., 2012; Jiang et al., 2014, 2016). The application of LBE fluorescence in the field of metal ion probe and biological imaging has not been reported. In this study, therefore, the green and cheap larch bark was chosen as the raw material to produce polymeric nanoparticles with AIE property. A time saving procedure was designed adopting 40% ethanol as the extraction solvent and ultrasonic crushing as the extraction method. The resulting polymeric nanoparticles owned good biocompatibility and potential both for cellular imaging and for the detection of ferric ions (Fe^3+^; Scheme 1). Our method for producing natural polymeric nanoparticles with AIE from the rational use of larch bark, could be regarded as a process turning waste into wealth.

## Results and Discussion

**Figure 1:**
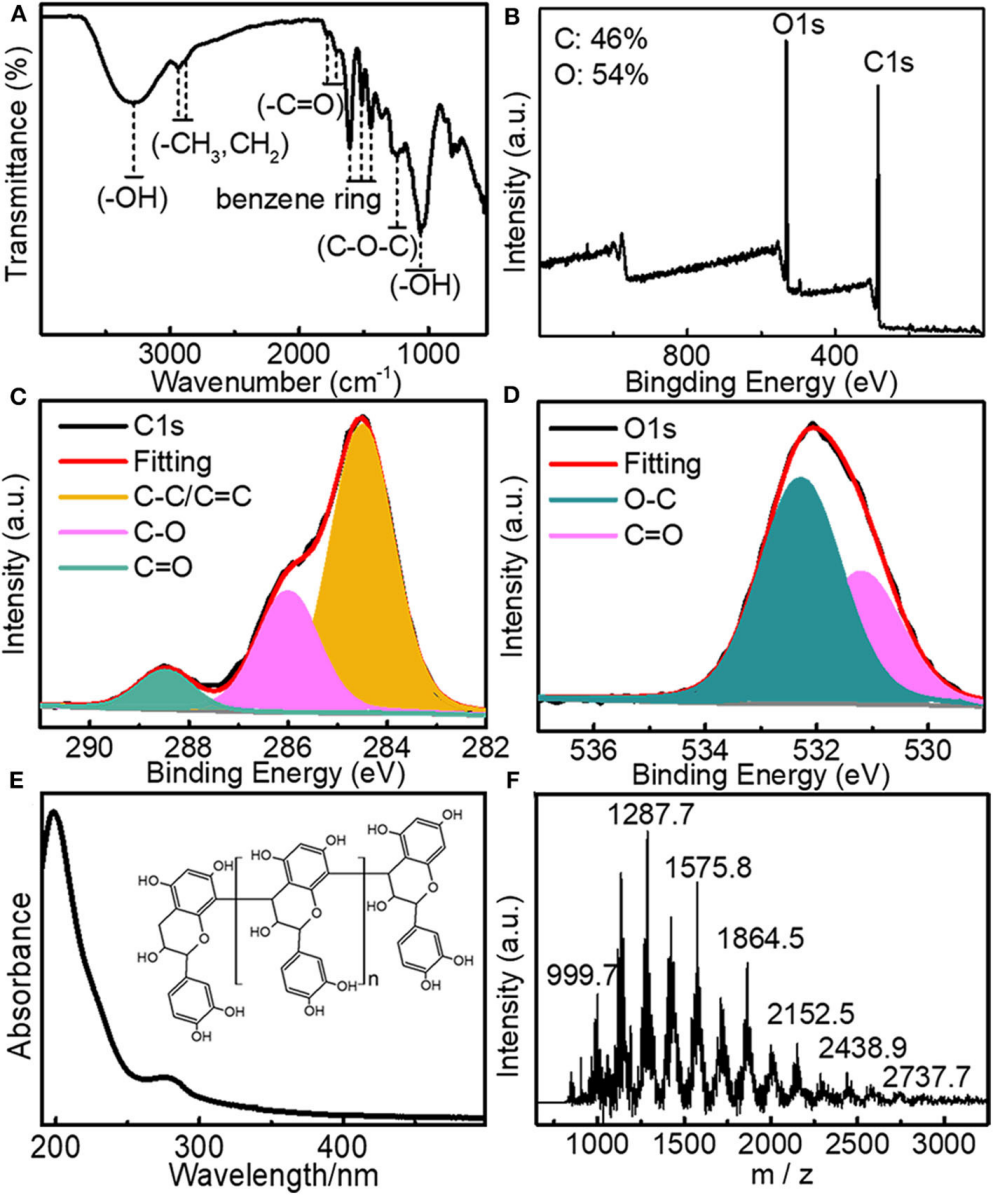
**(A)** FT-IR spectrum of LBE; **(B)** XPS spectrum of LBE; **(C)** High-resolution XPS C1s spectrum of LBE; **(D)** High-resolution XPS O1s spectrum of LBE; **(E)** UV-Vis spectrum of aqueous solution of LBE (10 μg/mL) (inset: structure of LBE); **(F)** Linear mode mass spectrogram showing molecular weight distribution of LBE.

Larch bark extracts (LBE) containing oligomeric proanthocyanidins was extracted from larch bark using the ultrasonic crushing method, with 40% aqueous ethanol as the extraction solvent and (Jiang et al., 2016; Luo et al., 2019), through dialysis (3,500 Dalton) and freeze-drying. The structure of LBE was analyzed by FT-IR spectroscopy, XPS, UV spectroscopy and mass spectrometry. The FT-IR spectrum (Figure 1A) illustrated that LBE contained hydroxyl groups (3,285 cm^−1^), -CH_3_/-CH_2_ groups (2,934 and 2,880 cm^−1^), benzene rings (1,608, 1,510 and 1,446 cm^−1^), -CH_3_ groups (1362 cm^−1^), carbonyl groups (1,784 and 1,710 cm^−1^), C-O-C bonds (1,246 cm^−1^) and aromatic hydroxyl groups (1,064 cm^−1^; Bao et al., 2015; Zhang et al., 2018). XPS analysis showed that LBE contained C (46%) and O (54%) elements (Figure 1B). Following analysis of the high resolution XPS spectra of elemental C (Figure 1C) and O (Figure 1D), the binding energies of 532.28, 286, and 284.5 eV were attributed to O-C, C-O, and C-C/C=C bonds, respectively (Sheng et al., 2011; He et al., 2018; Miao et al., 2018; Zhang et al., 2018). The UV absorption spectrum of LBE (Figure 1E) contained n-π^*^ and π-π^*^ transitions between 190 and 400 nm (Bi et al., 2018; Miao et al., 2018). The molecular weight distribution of LBE was analyzed by linear mode MALDI-TOF mass spectrometry after deionization and addition of Cs^3+^ as cationic reagent (Shoji et al., 2006; María et al., 2010; Jara and Josep, 2012). The primordial series of proanthocyanidins ion peaks (Figure 1F) showed a fixed difference of 288a. The difference between the observed value and the calculated value was compared using the equation m/z = 290 + 288a + 133 (Yang and Chien, 2000; Krueger et al., 2003), where 290 is the relative molecular mass of the terminal epicatechin unit, 288 is the relative molecular mass of extending epicatechin units, 133 is the relative atomic mass of Cs^+^ and a is the degree of polymerization. The analysis demonstrated that LBE is a mixture of oligomers proanthocyanidins (Figure 1E), ranging from trimer (m/z 999.7) to nonamer (m/z 2737.7), with the strongest ion peak for the tetramer (m/z 1287; Figure 1F), indicating that LBE mainly consists of tetramers proanthocyanidins.

**Figure 2:**
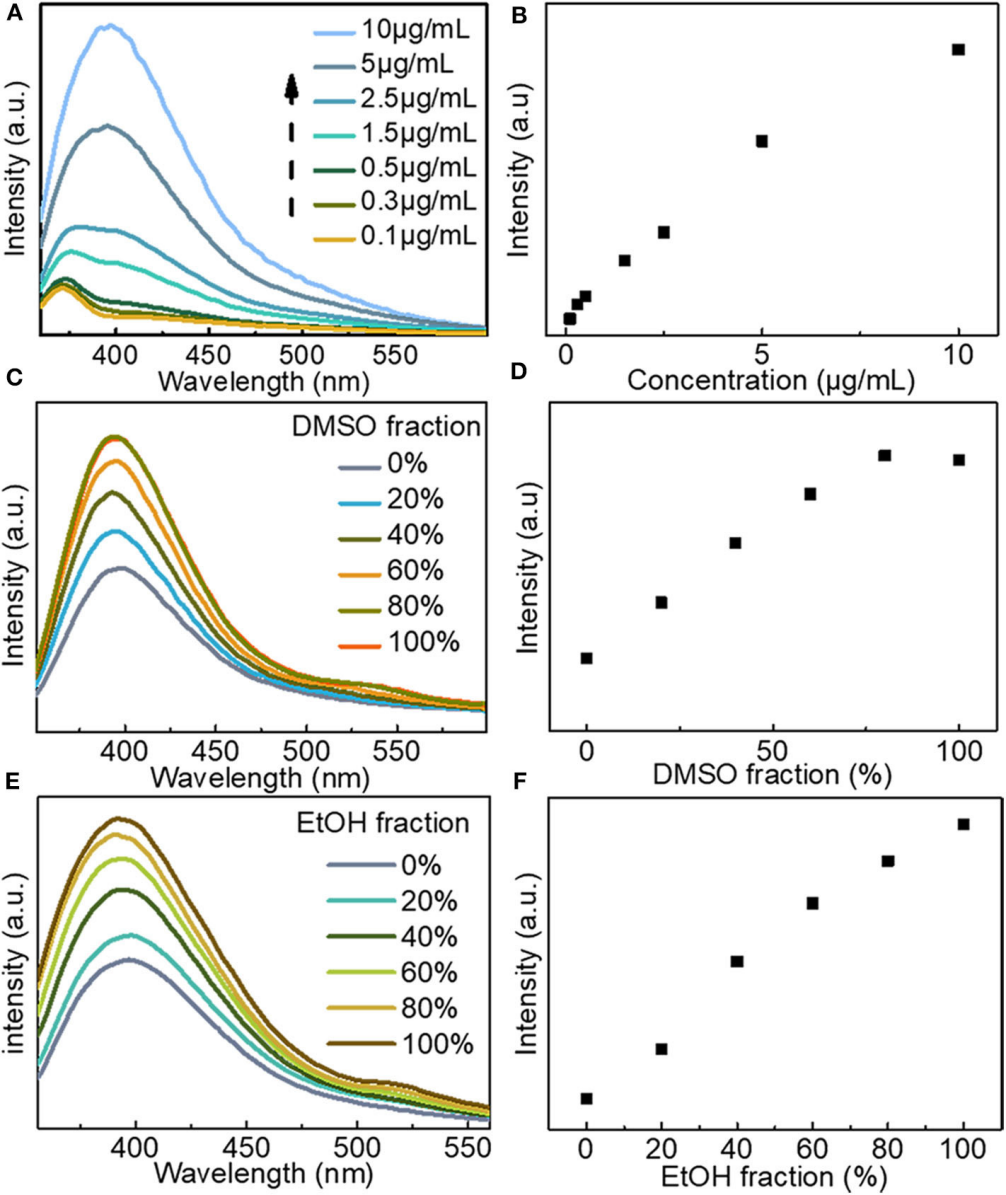
**(A)** Fluorescence emission spectra of LBE at different concentrations in water (Ex = 330 nm); **(B)** Fluorescence intensity of LBE at different concentrations in water; **(C)** Fluorescence emission spectra of LBE (15 μg/mL) in mixtures of DMSO and water (Ex = 330 nm); **(D)** Fluorescence intensity of LBE (15 μg/mL) in mixtures of DMSO and water (Ex = 330 nm); **(E)** Fluorescence emission spectra of LBE (10 μg/mL) in mixtures of EtOH and water (Ex = 330 nm); **(F)** Fluorescence intensity of LBE (10 μg/mL) in mixtures of EtOH and water (Ex = 330 nm).

Fluorescence analysis of LBE was carried out in water proven to be a good solvent. Measurement of fluorescence emission in an aqueous solution of LBE at different excitation wavelengths showed that the optimal excitation wavelength is 330 nm with an emission peak of 400 nm (Figure S1). Fluorescence emission of LBE exhibited excitation dependence. An aqueous solution of LBE displayed blue fluorescence irradiated with ultraviolet light (365 nm; Figure S2). The fluorescence intensity of LBE increased with the increasing concentration over the range from 0.1 to 10 μg/mL (Figures 2A,B). Ultraviolet absorption also increased and red shift with the increasing concentration (Figure S3). The enhanced fluorescence emission at higher concentrations occurred because the LBE molecules moved closer to each other, restricting rotation and vibration of the large aromatic rings in the molecules and thus resulting in the enhanced aggregation-induced fluorescence emission (Hu et al., 2014; Mei et al., 2014; He et al., 2018). The aggregation induced luminescence of LBE by mixing of good solvent (water) and poor solvent (DMSO and EtOH) was further studied. The results showed that the fluorescence emission intensity of the solution increased as the volume ratio of DMSO increased (Figures 2C,D). In the mixed system of water and DMSO, water clusters and hydrophobic DMSO clusters are formed in the mixed solvent (Zhang N. et al., 2013; Gujt et al., 2017). When the proportion of DMSO is increased, the hydrophilic LBE fluorophore molecules are aggregated in the mixed solvent by the hydrophobic DMSO clusters to form nanoparticles, which limits or restricts the intramolecular rotation of the LBE fluorophore, leading to the closing of non-radiative attenuation channels and the enhancement of fluorescence emission (Lu et al., 2012; Mei et al., 2014; He et al., 2018; Yang et al., 2019), also, it is the typical feature of the AIE phenomenon (Hong et al., 2009; Lu et al., 2012). Moreover, when the volume ratio of ethanol as poor solvent in the solvent increased, LBE also showed aggregation induced emission (AIE; Figures 2E,F).

**Figure 3:**
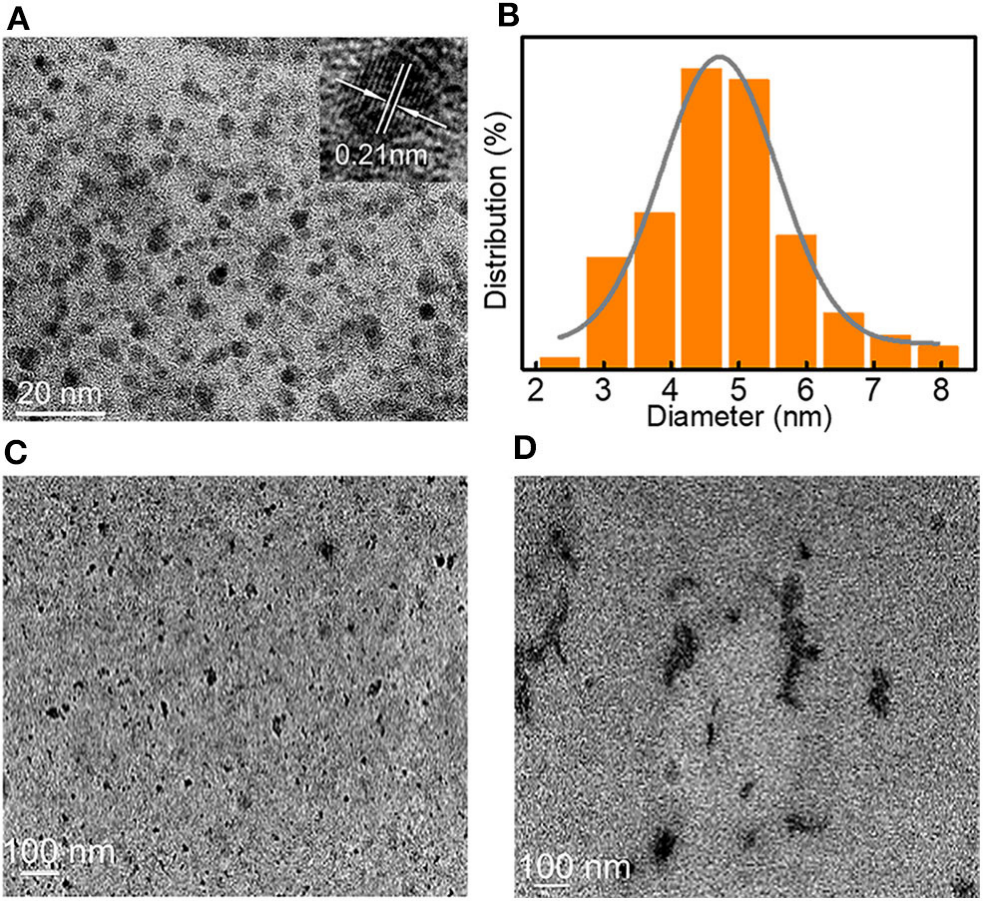
**(A)** TEM image of LBE in water (inset: HR-TEM image); **(B)** Particle size distribution of nanocrystalline LBE; **(C)** TEM image of LBE in DMSO; **(D)** TEM image of LBE in EtOH.

The luminescence mechanism was further investigated by high-resolution transmission electron microscopy (HR-TEM) of LBE dispersed in water (Figure 3). The TEM image showed a lattice spacing of 0.21 nm, which corresponded to the [100] facet of graphene-like structures (Chen et al., 2016). The particle size distributed in the range 2–8 nm, with an average diameter of ~4.5 nm (Figures 3A,B). The XRD pattern of LBE (Figure S4) illustrated an apparent peak at ~21.7°, which was attributed to an interlayer spacing of 0.34 nm, corresponding to the [002] facet of graphene-like structures (Zhu et al., 2013; Bi et al., 2018). The particles exhibited marked aggregation on addition of DMSO (Figure 3C) or ethanol (Figure 3D) to an aqueous solution, resulting in enhanced aggregate luminescence, which possessed luminescence characteristics similar to those of AIE (Hu et al., 2014; He et al., 2018). The photoluminescence quantum yield (PLQY) of LBE in water, DMSO and ethanol were 2.96, 3.25, and 3.72%, respectively.

**Figure 4:**
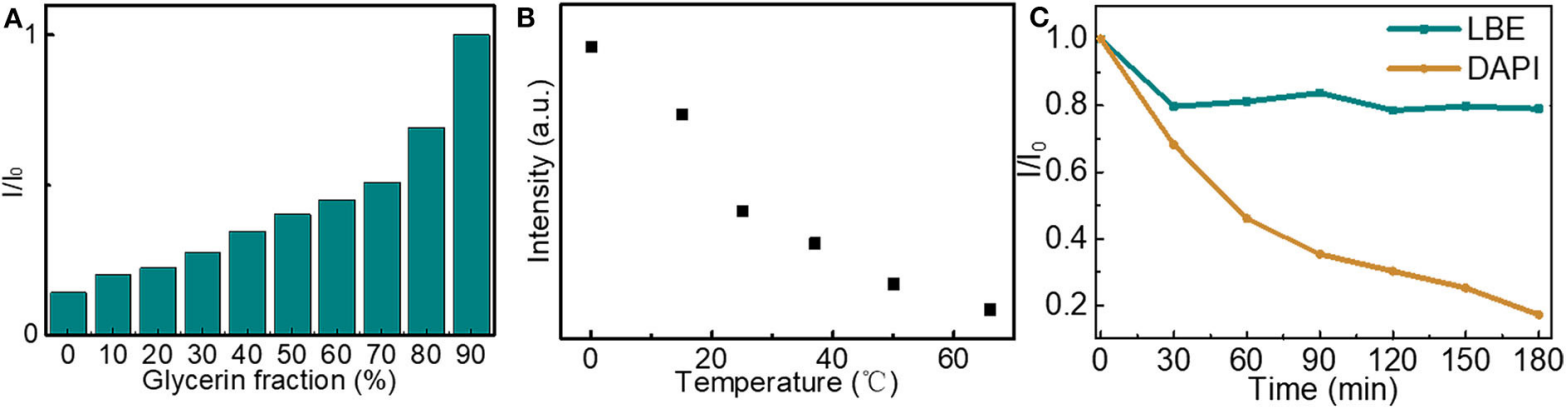
**(A)** Changes in fluorescence intensity of solutions of LBE (20 μg/mL) in different mixtures of water and glycerol (Ex = 330 nm); **(B)** Fluorescence intensity of aqueous solution of LBE (15 μg/mL) at different temperatures (Ex = 330 nm); **(C)** Fluorescence intensity of aqueous solutions of LBE (10 μg/mL, Ex = 330 nm) and DAPI (10 μg/mL, Ex = 365 nm) upon irradiation with UV light (365 nm, 200 mW cm^−2^). I is fluorescence intensity after UV irradiation and I_0_ is fluorescence intensity without UV irradiation.

To further investigate the luminescence properties of LBE, the effect of viscosity and temperature on the fluorescence intensity of LBE were analyzed. The fluorescence emission intensity of LBE increased with the increase of glycerol content when the water and glycerin were synergistically chosen to adjust solution viscosity with different volume ratios (Figure 4A and Figure S5), which mainly due to the high viscosity of the solvent limiting the rotation of the aromatic rings of LBE molecule, leading to an increment in the radiation transition, thus the LBE molecules show aggregation induced emission enhancement. The fluorescence intensity decreased with increasing temperature from 0 to 60°C (Figure 4B and Figure S6). The changes in fluorescence emission intensity with temperature occurred because the rotation of the macromolecular aromatic rings of LBE was restricted at lower temperatures, preventing non-radiative transitions and increasing fluorescence emission through radiative transitions (He et al., 2018). The resistance of LBE fluorescence to photobleaching was then investigated. The fluorescence intensity of an aqueous solution of LBE was reduced by about 20% after 30 min under strong ultraviolet light (200 mW/m^2^) and maintained further after continuous irradiation for 180 min (Figure 4C and Figure S7A). For comparison, the fluorescence intensity of the commercially available fluorescent stain 4′,6-diamidino-2-phenylindole (DAPI) was reduced by ~83% after irradiation for 180 min (Figure 4C and Figure S7B). It is worth noting that the intensity of the UV irradiance used here was 200 mW/cm^2^, which is 2,000-fold higher than the intensity used in a recent study (He et al., 2018). The fluorescence intensity of the LBE was stable, over the pH range 3.05–7.44 (Figure S8) of the intracellular microenvironments (Cosnier et al., 2013), suggesting that LBE was suitable for cellular imaging.

**Figure 5:**
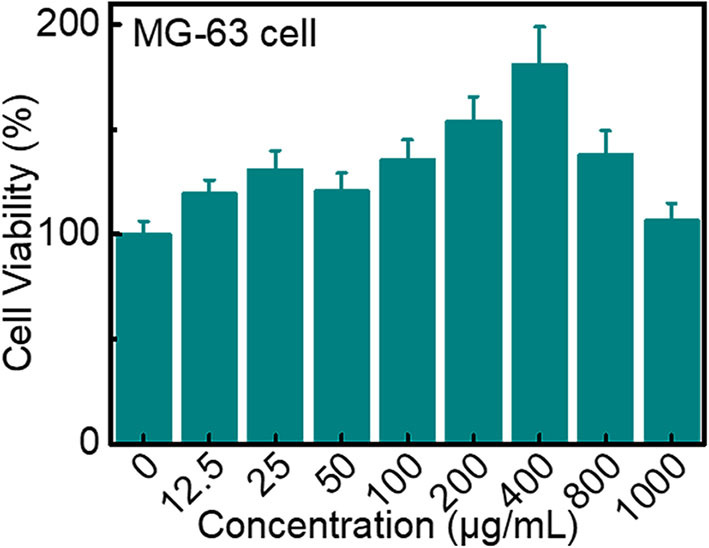
Viability of MG-63 cells after incubation with different concentrations of LBE for 24 h.

The toxicity of different concentrations of LBE toward MG-63 cells was investigated. LBE possessed growth effect on cells due to the good biological activity (Jiang et al., 2014, 2016). Incubation with LBE (1 mg/mL) for 24 h exhibited little effect on the survival of MG-63 cells, indicating that LBE was not toxic to MG-63 cells Figure 5. Flow cytometry for 3 day showed that the total apoptosis of MG-63 cells in the early and late stages was 9.2% (Q2+Q4; Figure S9), confirming that LBE was non-toxic.

**Figure 6:**
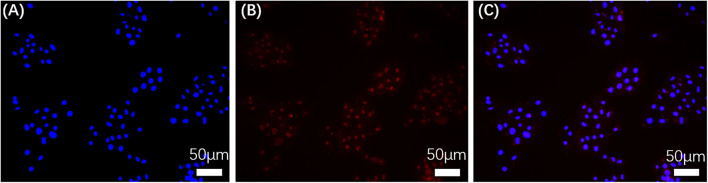
CLSM fluorescence images of MG-63 cells stained with DAPI and incubated with LBE for 10 h at 37°C. (**A**) Nuclei stained with DAPI; (**B**) Cells incubated with LBE; (**C**) Superposition of **(A,B)**.

Confocal laser scanning microscopy (CLSM) was utilized to monitor uptake of LBE by MG-63 cells over 10 h at 37°C (Figure 6). Figure 6A shows cell nuclei stained with DAPI and Figure 6B shows cells incubated with LBE nanoparticles, respectively. Figure 6C, which shows these two images superimposed, demonstrates that LBE nanoparticles efficiently crossed the cell membrane and became localized in the cell nucleus. Therefore, LBE nanoparticles can be employed as a fluorescent dye to stain nucleus.

**Figure 7:**
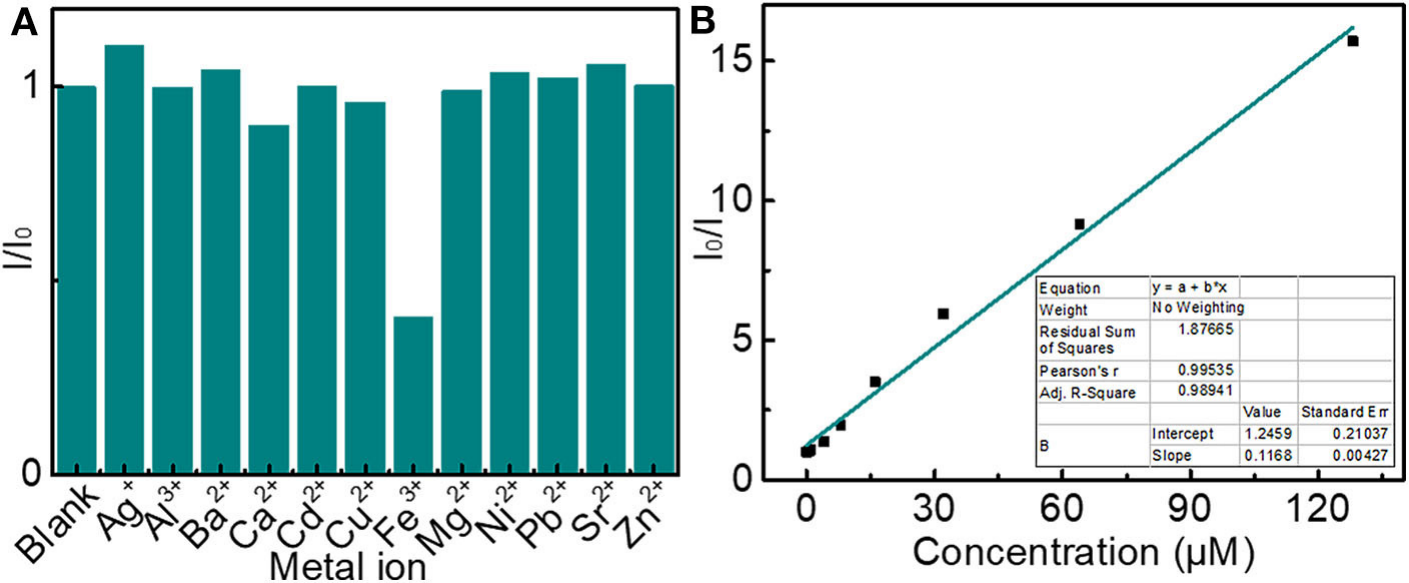
**(A)** Changes in fluorescence intensity of solutions of LBE (10 μg/mL) in the presence of different metal ions (5 μM); **(B)** Dependence of I/I_0_ on concentration of Fe^3+^ ions over the range 0–128 μM (inset: linear relationship of I/I_0_ vs. concentration of Fe^3+^ ions over concentration range 0–8 μM) (I_0_ and I are fluorescence intensity without and with ions, respectively).

The effect of different metal ions on the fluorescence intensity of an aqueous solution of LBE was investigated to explore the possible application of LBE in the detection of metal ions. Fe^3+^ ions were found to quench the fluorescence markedly, while the interference effect of other ions on fluorescence showed a slight or no effect (Figure 7A and Figure S10), which revealed that the LBE possessed a satisfying selectivity for the assay of Fe^3+^. Since the phenolic hydroxyl groups in LBE molecule could form complexes with Fe^3+^ ions as an electron donor, which was beneficial to facilitate charge transfer (Guo et al., 2015; Shen et al., 2017). The recombination of excitons was inhibited and then a significant fluorescence quenching occurred (Zhang Y. L. et al., 2013; Wang et al., 2018). Figure 7B and Figure S10 showed the relative fluorescence response of LBE(I_0_/I) according to the concentration of Fe^3+^, where I_0_ and I are the fluorescence intensities of LBE in the absence and presence of Fe^3+^ ions, respectively. The fluorescence quenching efficiency is described by Stern-Volmer plot (Sachdev and Gopinath, 2015; Arumugham et al., 2020). A good linear relationship with the correlation coefficient squared (R^2^) of 0.9894 was observed when the concentration of Fe^3+^ changed from 0 to 128 μM (Figure 7B). The limitation of detection (LOD) was estimated to be 0.17 μM based on the signal-to-noise ratio (SNR) of 3 (3σ/m, σ is the standard deviation of the blank signal (*n* = 3) and m is the slope of the linear fit). This value is well below the maximum allowable level of Fe^3+^ in drinking water set by the World Health Organization (5.36 μM), Moreover, the method of detecting Fe^3+^ based on carbon dots in this article was comparable or even better than that in other previous reports (Shen et al., 2017; Chen et al., 2019; Arumugham et al., 2020). The results indicated that LBE owned a good application prospect in the detection of trace Fe^3+^ due to the low LOD of Fe^3+^ (Wang et al., 2018).

## Conclusions

Larch bark is a natural biomass resource typically regarding as waste. Here, we used a simple method to convert larch bark into LBE nanoparticles, which showed aggregation-induced luminescence in solution. Enhancement of fluorescence emission was observed at high concentrations, in poor solvents, at low temperatures and in highly viscose solvents. Solutions of LBE were resistant to photobleaching under strong UV irradiation. Notably, LBE possessed good biocompatibility and exhibited no obvious toxicity to MG-63 cells, even at a high concentration (1 mg/mL). LBEcould be used as a biological chromogenic fluorescent staining agent for cell nuclei. Additionally, Fe^3+^ ions were found to own a marked quenching effect on the fluorescence of LBE solutions. The limitation of detection (LOD) was 0.17 μM so that LBE possessed a good application prospect in the detection of trace Fe^3+^.

## Methods

### Preparation of LBE

Larch bark powder (3 g) was extracted with 40% aqueous ethanol (60 mL) for 25 min at 50–55°C using scientz-1,200e ultrasonic cell grinder, operating at 500 W. The extract was separated by centrifugation and the larch bark was re-extracted as described above. The combine two extraction solutions that the combined extracts were dialyzed with deionized water (1 L) for 3 days under magnetic stirring using a dialysis bag (MWCO: 3500 Da). The external dialysate was concentrated and freeze-dried to give LBE as a brown solid.

### Characterization

The extraction is carried out by scientz-1,200e ultrasonic cell grinder. TEM and HR-TEM images were collected using a JEM-2,100 transmission electron microscope. X-ray photoelectron spectroscopy (XPS) was carried out using an Escalab 250Xi X-ray photoelectron spectrometer. FTIR spectra were recorded using a Frontier Fourier transform infrared spectrometer. UV-vis absorption spectra were recorded using a TU-1950 ultraviolet-visible spectrofluorometer. Photoluminescence (PL) measurements were carried out using an LS55 fluorescence spectrometer. Fluorescence decay curves were measured using a DeltaFlex modular fluorescence lifetime instrument. PL quantum yields were measured using an FLS1000 fluorescence spectrometer. Fluorescence images were captured using a DMI4000 B inverted fluorescence microscope. The molecular weight distribution of LBE was measured using an AutoflexIII mass spectrometer.

### Cytotoxicity Test

The effects of different concentrations of LBE on the viability of MG-63 cells were determined using a Cell Counting Kit-8 (CCK-8) assay. Cell suspensions, harvested at the exponential growth phase of the cells, were plated onto a 96-well plate at a density of 5,000 cells per well. The cells were then grown overnight at 37°C in culture medium (10% FBS + 90% DMEM/F12 + 100 μg/mL Normocin) in a humidified atmosphere of 5% CO_2_ to ensure that the cells adhered to the orifice plates. The wells were then divided into a blank control group (medium only), a negative control group (medium + cells) and 8 test groups (medium + cells + LBE), with 8 wells in each group. In the test groups, the cells were cultured in the presence of different concentrations (0, 12.5, 25, 50, 100, 200, 400, 800, 1,000 μg/mL) of LBE. The cells were placed in an incubator at 37°C in a humidified atmosphere of 5% CO_2_ for 24 h. CCK-8 (10 μL) was then added to each well and the cells were incubated for a further 4 h at 37°C. The absorbance of each well was measured at 450 nm using a Multiskan GO microplate reader (Thermo Fisher Scientific, Vantaa, Finland). Cell viability was defined as the ratio of absorbance in the presence of LBE to that in the absence LBE.

(1) $$\begin{array}{l} \text{Cell viability }\left(\%\right)\\ =\;\frac{OD_{test}-OD_{blank\;control}}{OD_{negative\;control}-OD_{blank\;control}}\;\times \;100\% \end{array}$$

### Cellular Imaging

MG-63 cells were inoculated into 48-well plates (10,000 cells per well) with clean cover glasses. The cells were then grown for 24 h at 37°C in culture medium (10% FBS + 90% DMEM/F12 + 100 μg/mL Normocin) in a humidified atmosphere of 5% CO_2_. LBE was added and the cells were cultured in a saturated humidity incubator at 37°C under an atmosphere of 5% CO_2_ for 10 h. The cover glasses were removed and washed three times with PBS. The cells were immobilized with pre-cooled 4% paraformaldehyde solution for 30 min and then dyed using a solution of DAPI in PBS (10 μg/mL) for 5 min. After each operation, the cover glasses were washed three times with PBS. The cover glasses were sealed with anti-fluorescence quenching agent and images were captured using an inverted fluorescence microscope.

### Sensing of Fe^3+^ and Other Ions

Three mL of LBE solution with concentration 10 μg/mL was put into a four-way quartz cuvette with a lid, then added 20 μL of Fe^3+^ solution with different concentrations by means of a pipette to make the Fe^3+^ concentration range from 0 to 128 μL. All the obtained solutions were shaken evenly, and then the fluorescence emission spectrum (Ex = 330nm) was measured after standing for 1 min. Similarly, other metal ions were also analyzed in this way and they were newly prepared like the Fe^3+^ solution of different concentrations.

### Materials and Reagents

Larch bark was obtained from deciduous pines of the Daxing'an Mountains, Heilongjiang Province, China. All reagents were analytical reagent grade and were used as received without further purification. Deionized water was prepared using a Clever-Q30 UT water filtration system (Zhiang Instrument Co., Ltd. Shanghai, China). A Cell Counting Kit-8 (CCK-8) assay kit was purchased from Dojindo Laboratories (Kumamoto, Japan) and Normocin was purchased from InvivoGen (San Diego, CA, USA). Fetal bovine serum (FBS), Dulbecco's modified Eagle's medium/Ham's F-12 medium (DMEM/F12) and phosphate buffered saline (PBS) were Gibco reagents and were purchased from Thermo Fisher Scientific Inc. (Waltham, MA, USA). MG-63 cells was purchased from the Chinese Academy of Sciences (Shanghai, China).

## Data Availability Statement

All datasets generated for this study are included in the article/Supplementary Material.

## Author Contributions

JN and YH conducted the extract experiments. ZP performed the cell imaging experiments. SH, MG, and GJ carried out characterization and analysis of the samples. SH and CZ wrote the manuscript and made substantial revisions. Supervision, funding acquisition, review, and editing of the manuscript were carried out by SL and JC. All authors have approved the final version of the manuscript.

## Conflict of Interest

The authors declare that the research was conducted in the absence of any commercial or financial relationships that could be construed as a potential conflict of interest.
