# Supplementary material for: Biomass-Based Polymer Nanoparticles With Aggregation-Induced Fluorescence Emission for Cell Imaging and Detection of Fe3+ Ions
Source: Front Chem. 2020 Jul 3;8:563. doi: 10.3389/fchem.2020.00563 (PMC7350900; doi:10.3389/fchem.2020.00563)
Supplement: Supplementary file 1 [file Table_1.docx]

Supplementary Material

**
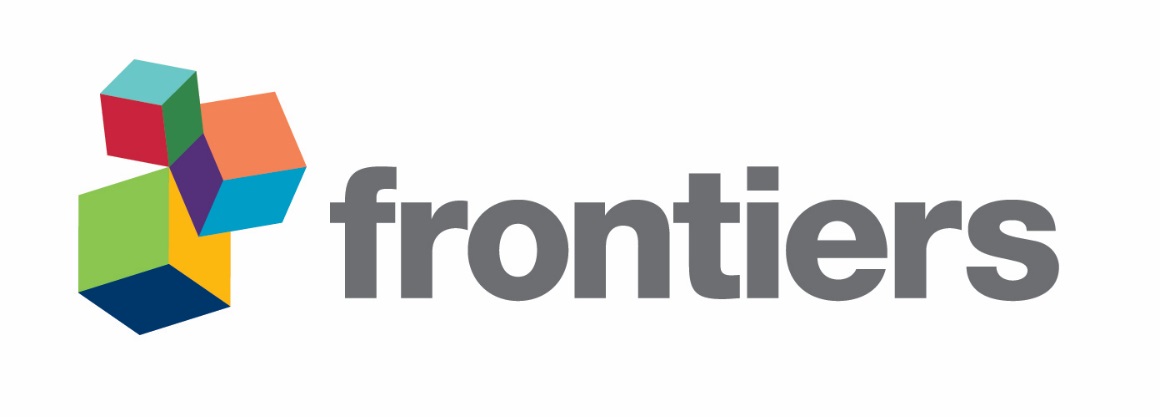
**


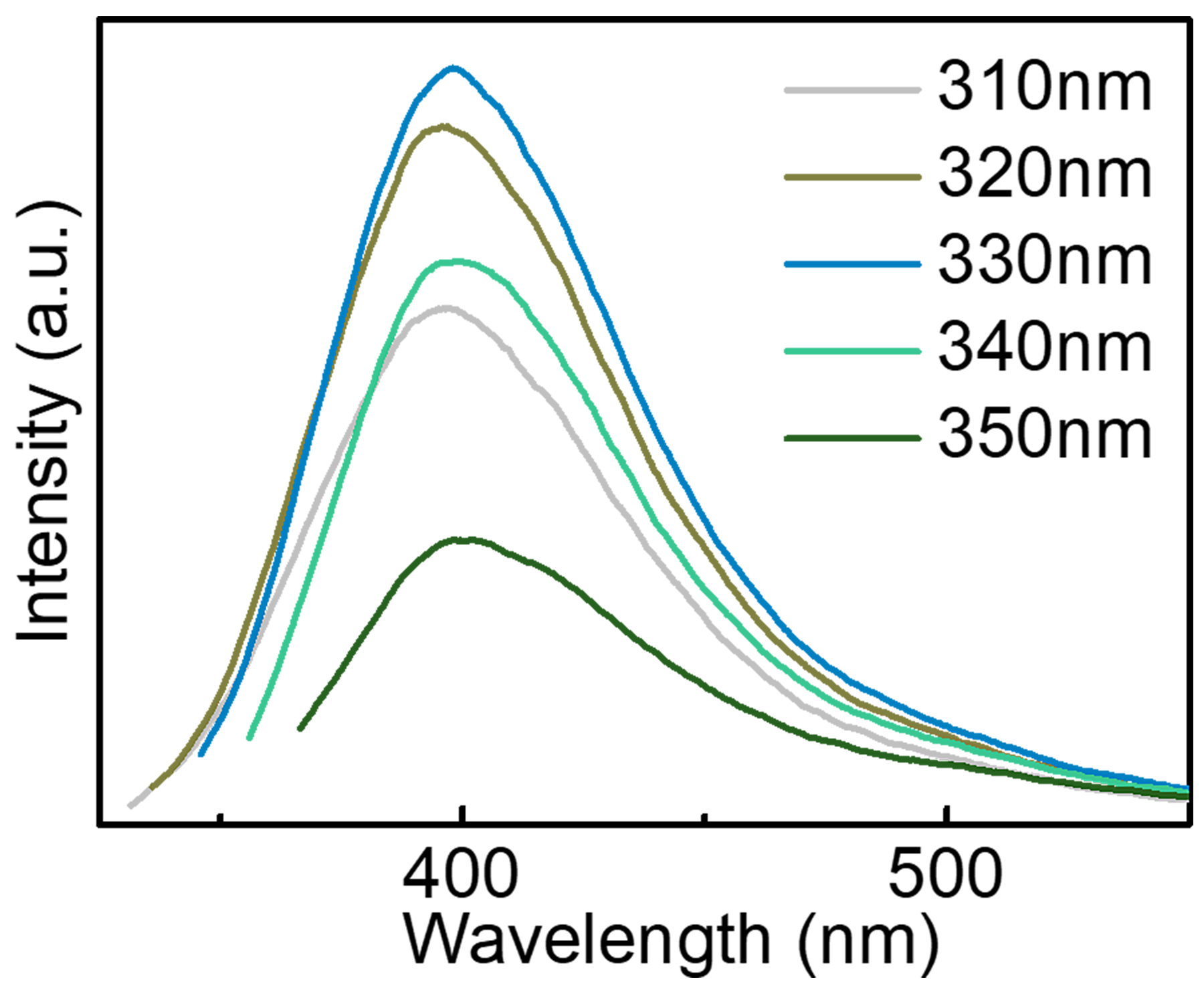


**FIGURE S1** Fluorescence emission spectra of aqueous solution of LBE at different excitation wavelengths


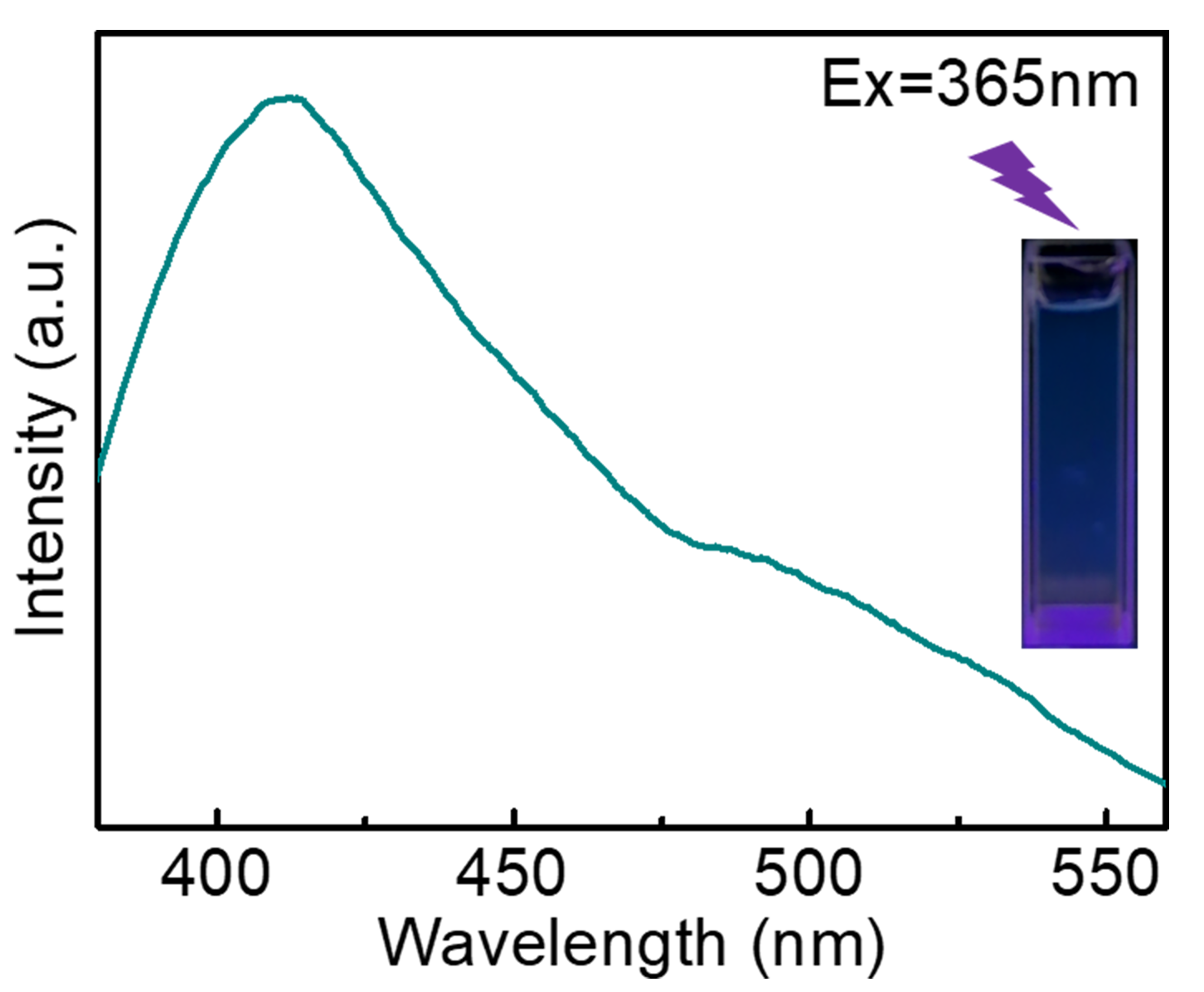


**FIGURE S2** Fluorescence emission spectrum of aqueous solution of LBE (Ex=365 nm); Inset: image showing aqueous solution of LBE irradiated at 365 nm


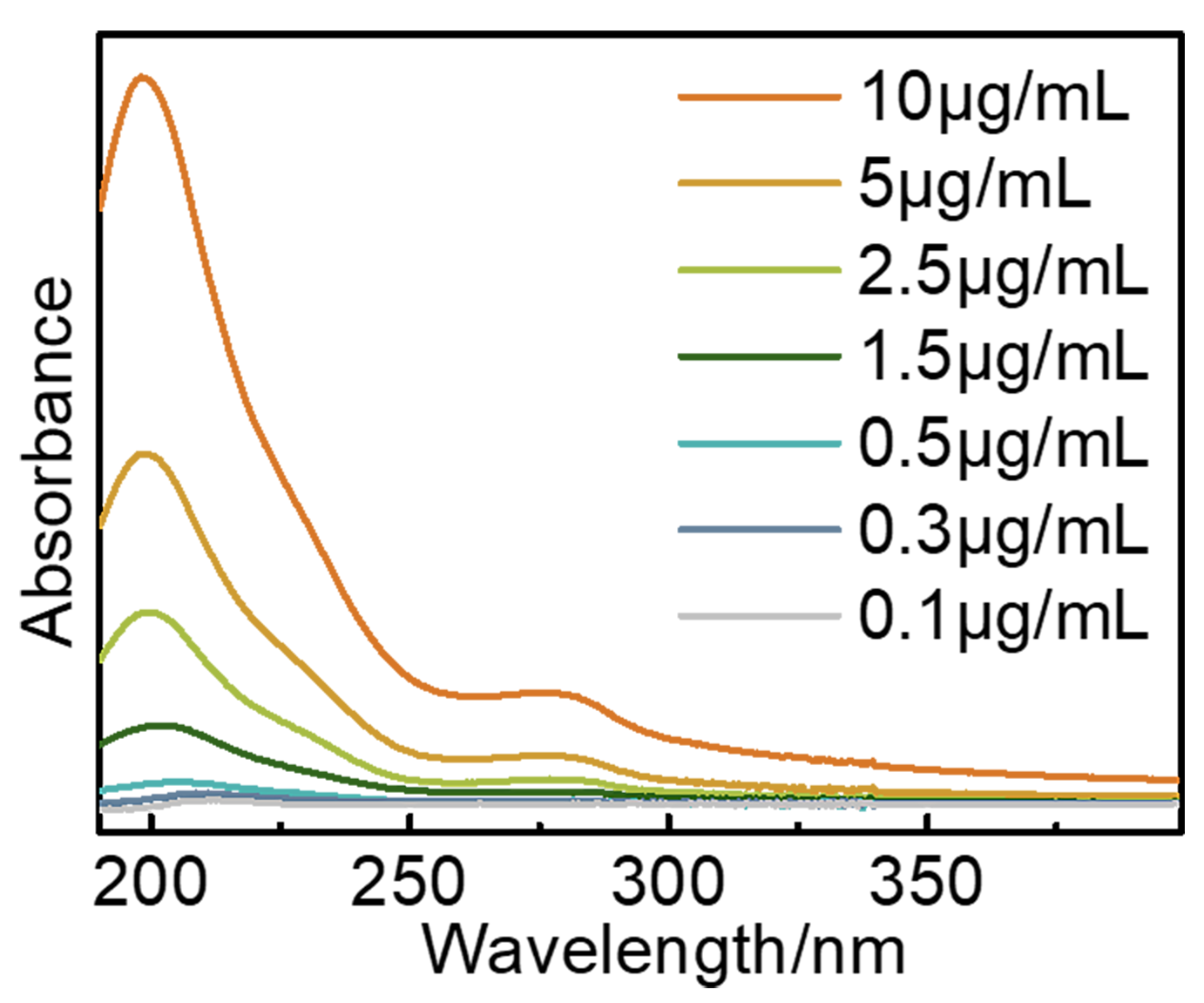


**FIGURE S3** UV-vis spectra of aqueous solutions of LBE with different concentrations


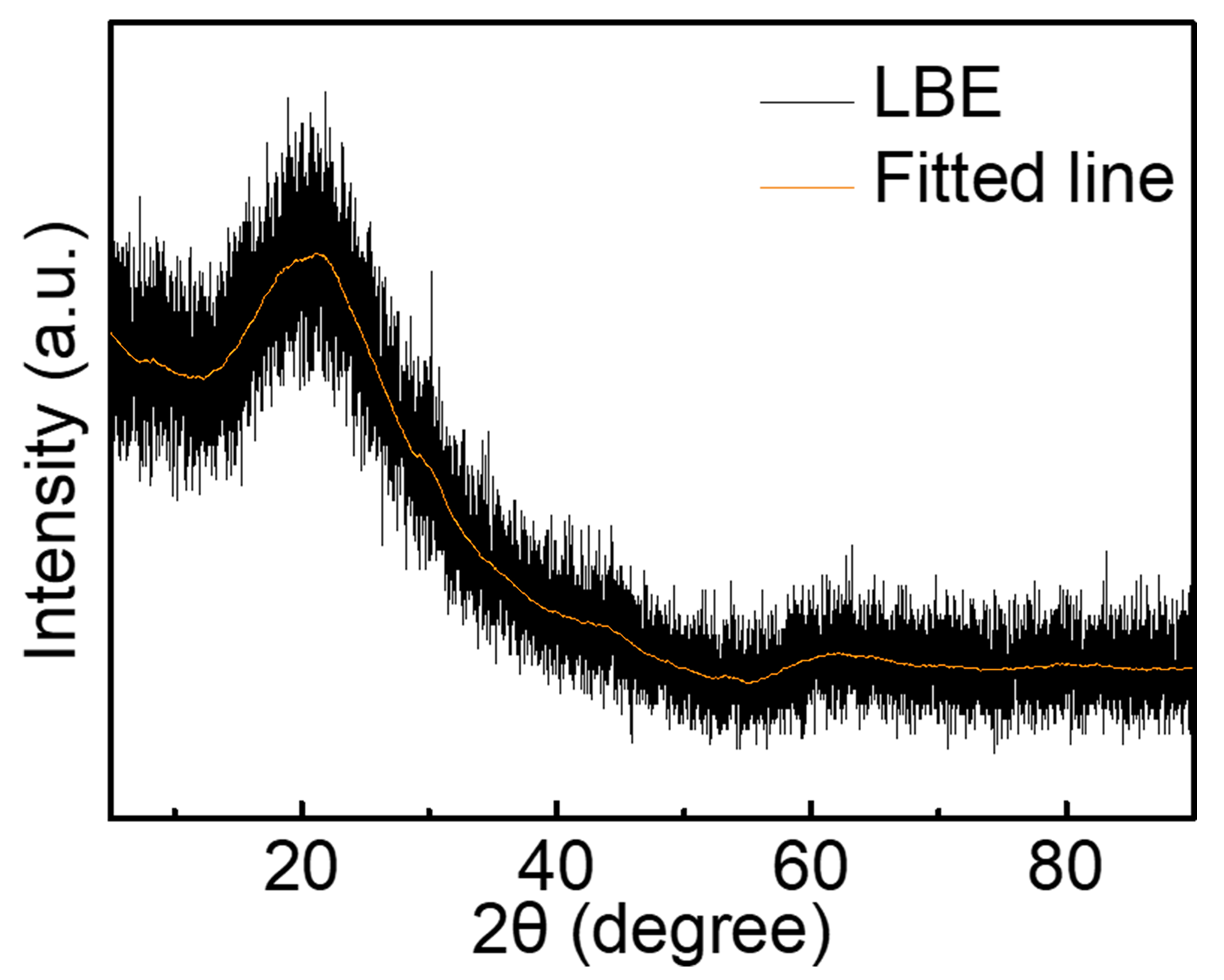


**FIGURE S4** XRD spectrum of LBE


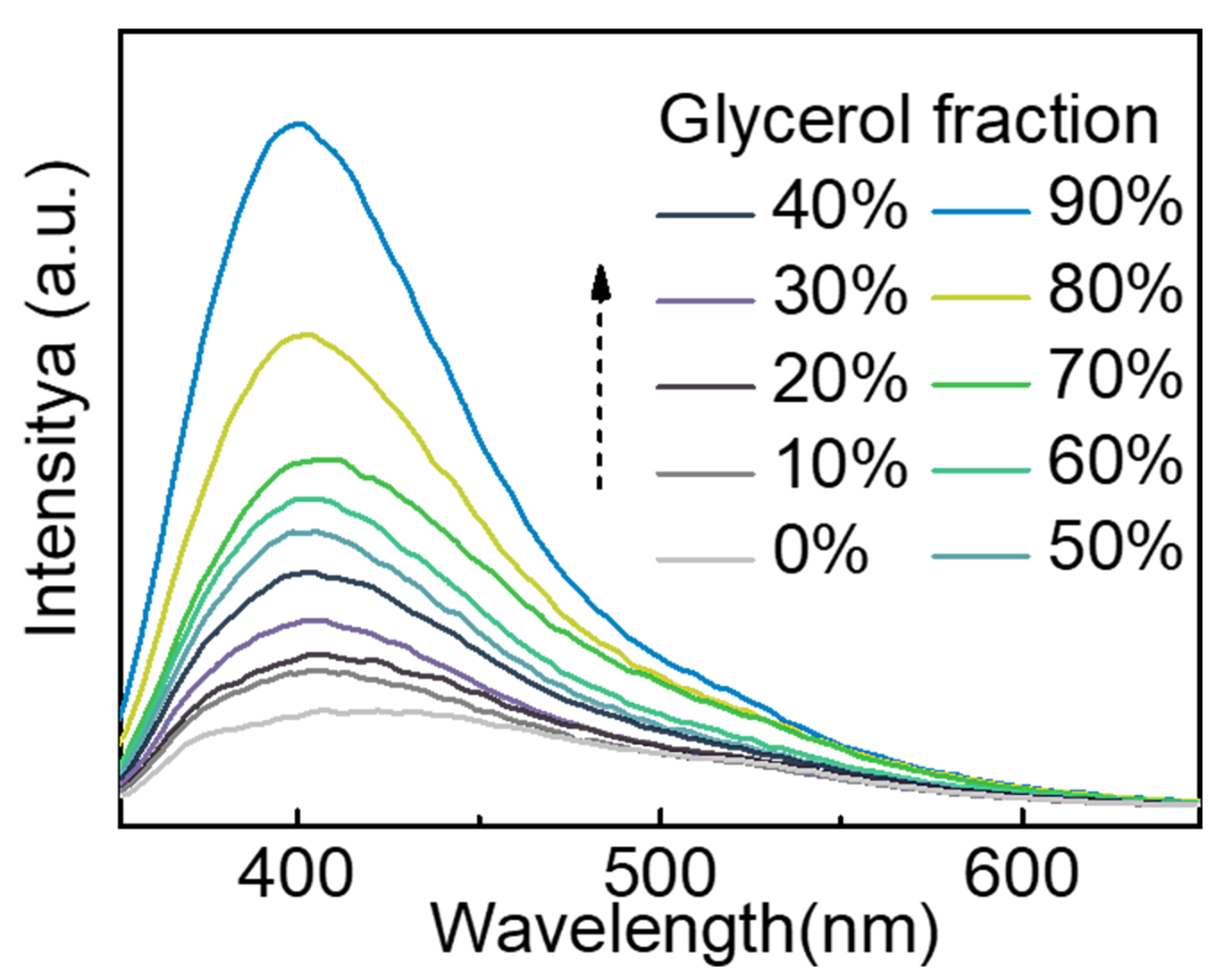


**FIGURE S5** Fluorescence emission spectra of solutions of LBE (20 μg/mL) in different mixtures of water and glycerol (Ex=330nm)


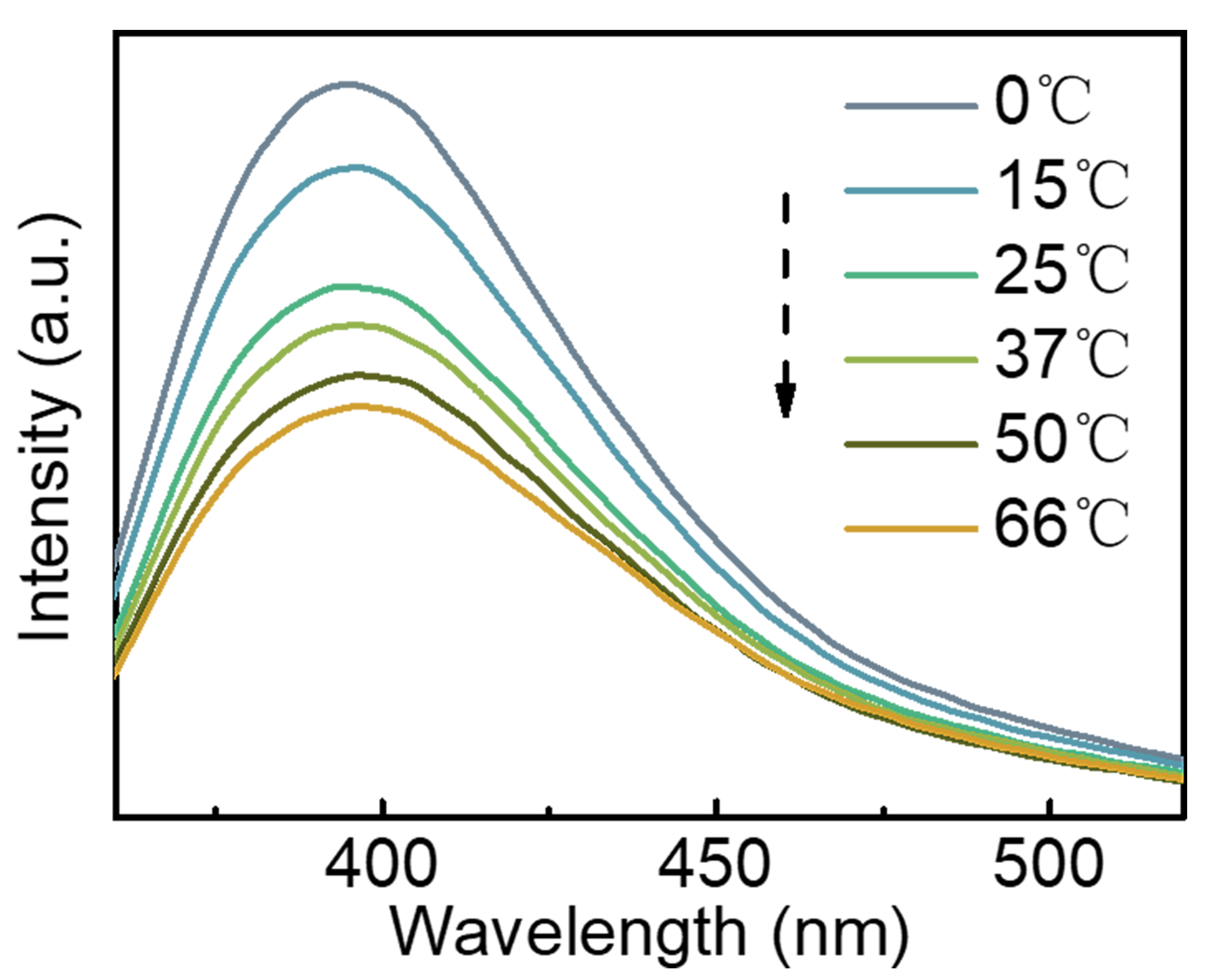


**FIGURE S6** Fluorescence emission spectra of aqueous solution of LBE (15 μg/mL) at different temperatures (Ex=330nm)


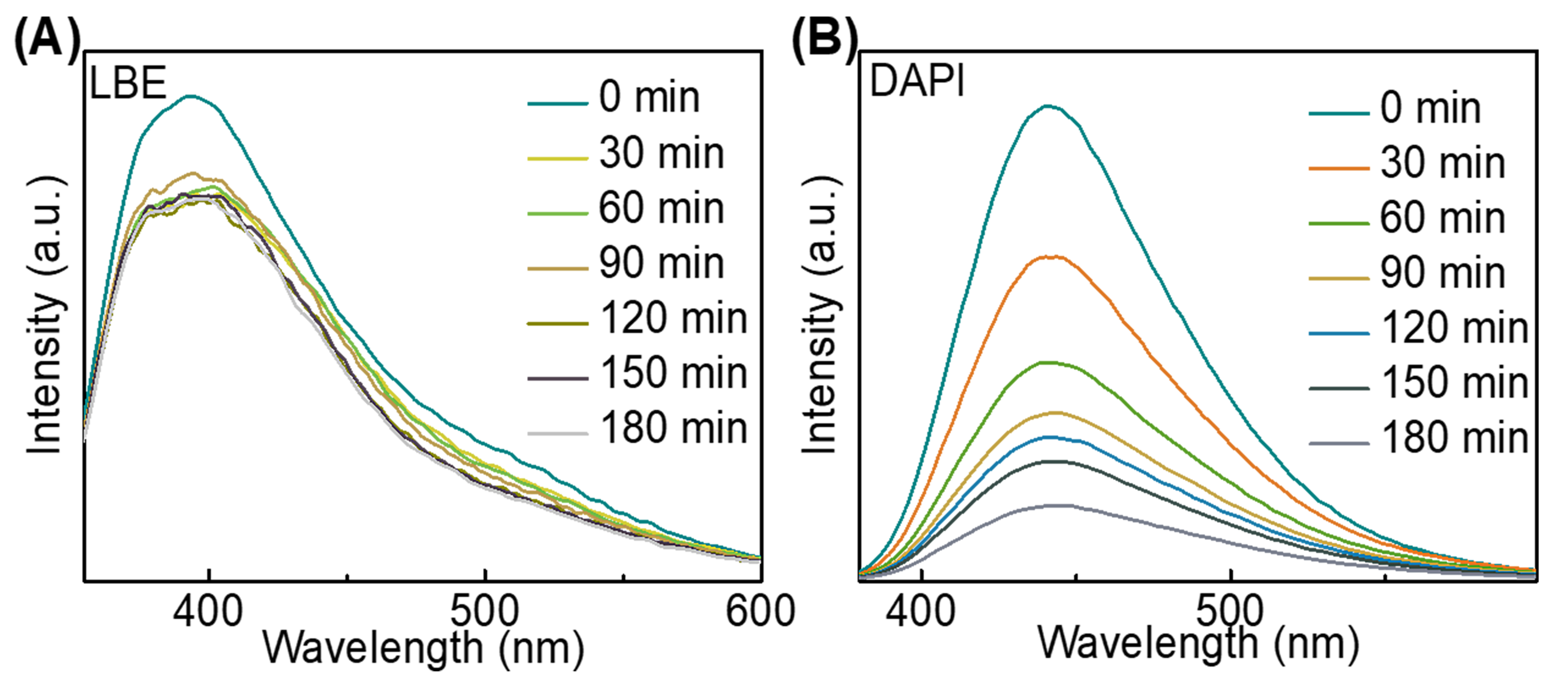


**FIGURE S7** Fluorescence emission spectra of aqueous solutions of (A) LBE (10 μg/mL) and (B) DAPI (10 μg/mL) upon ultraviolet irradiation (365 nm, 200 mW cm^-2^) for different lengths of time


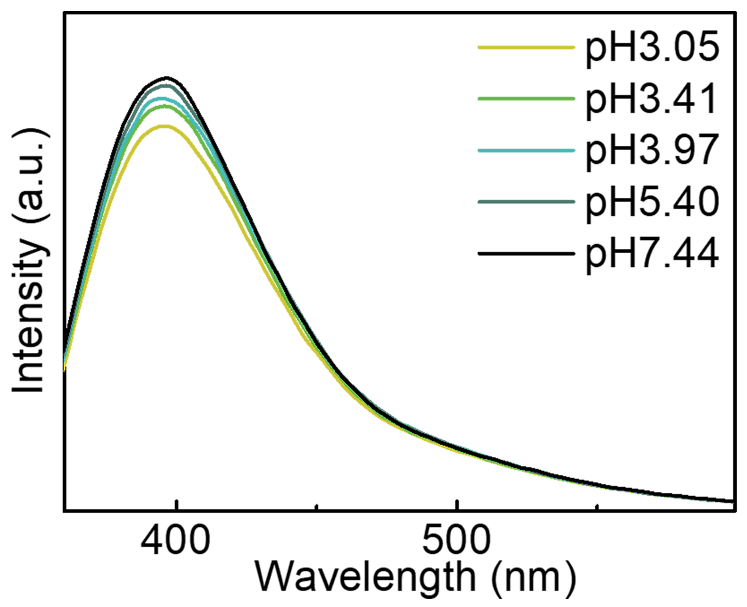


**FIGURE S8** Fluorescence emission spectra of aqueous solution of LBE (20 μg/mL) at different pH (Ex=330nm)


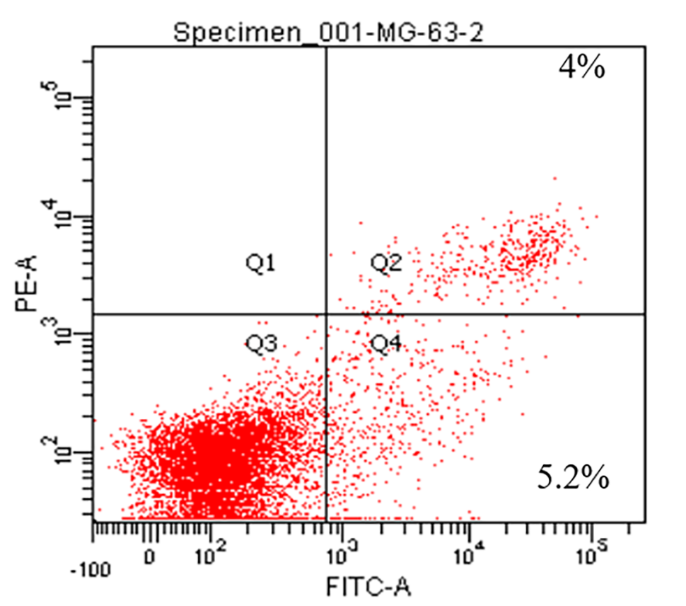


**FIGURE S9** Cytotoxicity evaluated by flow cytometry using MG-63 cells treated with LBE (100 μg/mL) for 72 h.


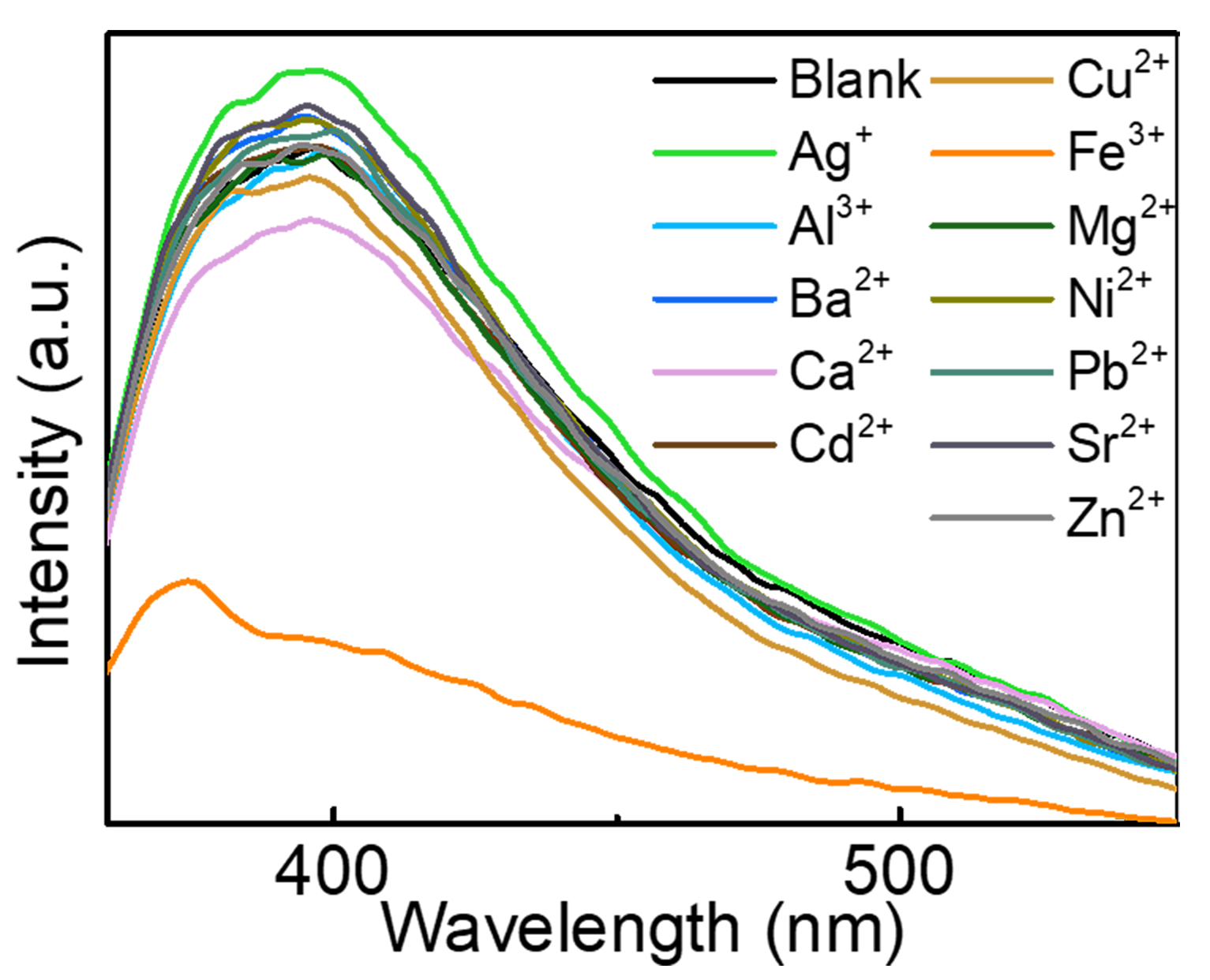


**FIGURE S10** Fluorescence emission spectra of aqueous solutions of LBE (10 μg/mL) in the presence of different metal ions (5 μM) (Ex=330nm)


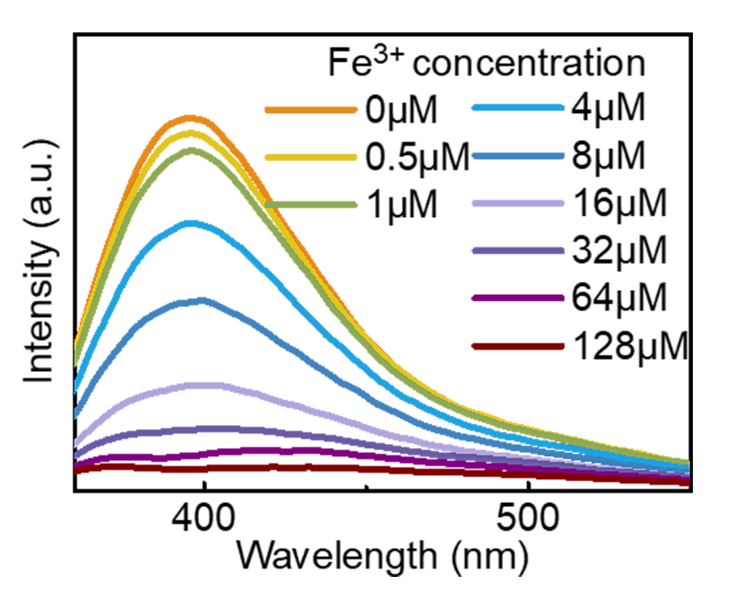


**FIGURE S11** Fluorescence emission spectra of aqueous solutions of LBE (20 μg/mL) in the presence of different concentrations of Fe^3+^
